# Sketches of chimpanzee (*Pan troglodytes*) *hoo*’s: vowels by any other name?

**DOI:** 10.1007/s10329-023-01107-3

**Published:** 2023-12-18

**Authors:** Axel G. Ekström, Jens Edlund

**Affiliations:** Division of Speech, Music and Hearing, KTH Royal Institute of Technology, Stockholm, Sweden

**Keywords:** Speech acoustics, Articulatory phonetics, Vowel quality, Primatology

## Abstract

In human speech, the close back rounded vowel /u/ (the vowel in “boot”) is articulated with the tongue arched toward the dorsal boundary of the hard palate, with the pharyngeal cavity open. Acoustic and perceptual properties of chimpanzee (*Pan troglodytes*) *hoo*’s are similar to those of the human vowel /u/. However, the vocal tract morphology of chimpanzees likely limits their phonetic capabilities, so that it is unlikely, or even impossible, that their articulation is comparable to that of a human. To determine how qualities of the vowel /u/ may be achieved given the chimpanzee vocal tract, we calculated transfer functions of the vocal tract area for tube models of vocal tract configurations in which vocal tract length, length and area of a laryngeal air sac simulacrum, length of lip protrusion, and area of lip opening were systematically varied. The method described is principally acoustic; we make no claim as to the actual shape of the chimpanzee vocal tract during call production. Nonetheless, we demonstrate that it may be possible to achieve the acoustic and perceptual qualities of back vowels without a reconfigured human vocal tract. The results, while tentative, suggest that the production of *hoo*’s by chimpanzees, while achieving comparable vowel-like qualities to the human /u/, may involve articulatory gestures that are beyond the range of the human articulators. The purpose of this study was to (1) stimulate further simulation research on great ape articulation, and (2) show that apparently vowel-like phenomena in nature are not necessarily indicative of evolutionary continuity per se.

## Introduction

Great apes, such as chimpanzees (*Pan troglodytes*) (Plooij et al. 2015; Grawunder et al. 2022) and orangutans (*Pongo* spp.) (Lameira and Wich 2008; Ekström et al. 2023), produce hoot-like calls with a vowel-like quality that seems to be similar to that of human back vowels. In human speech, vowels such as /u/ (the vowel in “boot”) are articulated with the body of the tongue close to the hard palate and the pharyngeal cavity open. However, there are likely substantial limitations to the tongue and jaw morphology of nonhuman primates that preclude them from making similar movements (Lieberman et al. 1972; Takemoto 2008; De Boer and Fitch 2010; see also Ekström 2023a), with morphological analyses of the chimpanzee tongue suggesting it has the most degrees of freedom in protrusion and retrusion rather than in anterior stretching inside the oral cavity (Takemoto 2008). In addition, all nonhuman mammals have a short, narrow pharynx, while in humans there has been reconfiguration of the vocal tract during evolution, with reorganization of the cranium, reconfiguration of the airways, a permanently descended tongue root and larynx, expansion of the pharyngeal cavity, and rounding of the tongue (Negus 1949; Laitman et al. 1978; Laitman and Heimbuch 1982; Lieberman 1984, 2012; De Boer and Fitch 2010; Iwasaki et al. 2019; Ekström and Edlund 2023), which have allowed humans to acquire greater degrees of freedom in tongue movement.

While three recent studies purportedly show that limitations to speech in nonhuman primates have been overstated, all have significant limitations. The vowel space of the purportedly “speech-ready” macaque presented by Fitch et al. (2016) was only partially based on data from actual vocalizations, with much of the space based on outlier data of extreme mandibular contortions observed while the animal was yawning, which led to an unrealistic comparison with human vowel space (Everett 2017). Even allowing for this inflated articulatory space, monkey phonetic range did not extend to /u/ (Lieberman 2017; Ekström 2023b). Secondly, while Boë et al. (2017) claimed that observed baboon (*Papio papio*) “proto-vocalic” properties indicated lingual capabilities, this was conjecture based only on the data to be explained. A more parsimonious explanation is that would-be intra-vocalic properties are achieved by exploiting differences in prognathic (long-faced) jaw opening (Fant 1960; Lindblom and Sundberg 1969), as in the meows of domestic cats (*Felis catus*) (see Ekström 2023c). Finally, while values reported for chimpanzee call properties by Grawunder et al. (2022) purportedly show an “expansion of vowel-like space,” these data were likely biased by linear-predictive coding procedures that mistook harmonic partials for resonance frequencies (Ekström 2023d). The results of these studies do not constitute serious challenges to the claim that nonhuman primate lingual articulation is limited by these species’ anatomy compared with that of a human. While the /u/-like calls reported for *hoo’s* likely do not suffer from this problem, the articulatory correlates reported by the authors illustrate that vocalizing chimpanzees achieve these qualities with characteristic lip protrusion and lip rounding. There is, however, no evidence that these qualities are achieved using lingual gestures as in humans. Accordingly, overlapping vowel-like qualities may in reality reflect highly disparate articulatory gestures. This is significant for any implications for the evolution of speech or related capacities (Grawunder et al., 2022).

**Fig. 1:**
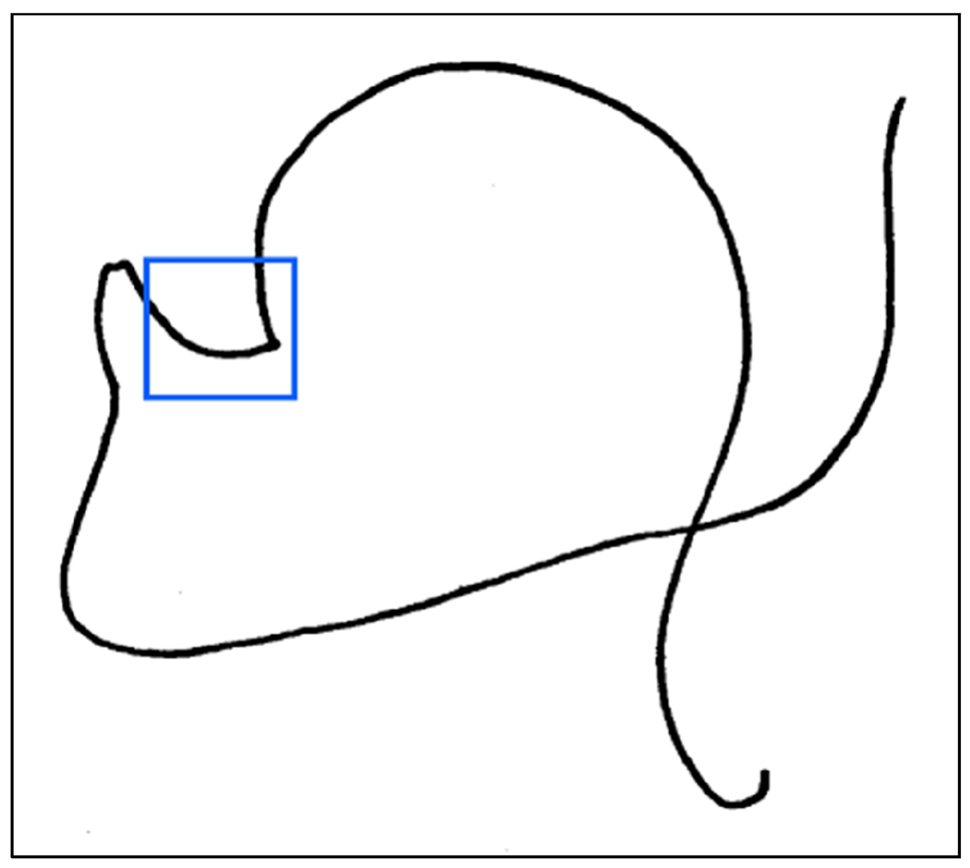
Tongue shape relative to mandible for pronunciation of the Swedish /u/ [from the articulatory model by Lindblom and Sundberg (1969)]. Retraction of the tongue tip and blade creates a pocket anterior to the mandibular teeth (*blue*). Lieberman et al. (1972) argued that, among other morphological characteristics, the simian shelf precludes articulation of the human /u/ by chimpanzees. [Image adapted from Lindblom and Sundberg (1969) with permission]

While in human speech the first formant frequency (*F*_1_) is typically considered to correspond to the resonance of the front cavity (i.e., the opening of the jaw or height of the tongue), and the second formant frequency (*F*_2_) to the resonance of the “back” or pharyngeal cavity, nonhuman primates, which essentially lack a posterior cavity, are likely incapable of comparable articulation to achieve the qualities of human vowels (Lieberman 1984, 2012; Takemoto 2008; De Boer and Fitch 2010; Fitch et al. 2016). In addition, chimpanzees possess a boney horizontal ridge that projects inward from the inside of the mandible and effectively creates a bony thickening, which is known as the simian shelf. From their analysis of chimpanzee lingual and oral anatomy, Lieberman et al. (1972, p. 297) argued that “The vowel /u/ is virtually impossible for the chimpanzee to articulate. A large front cavity requires the mandible to be lowered because the simian shelf prevents the tongue body motion found in man. However, the required lip rounding is incompatible with a lowered mandible.” (Fig. 1). Furthermore, cineradiographic images of vocalizing nonhuman primates suggest that the tongue is not actively employed in articulation by simians (Fitch 2000). Thus, the acoustic properties of chimpanzee *hoo*’s (see Grawunder et al. 2022) present researchers with an intriguing question: given an “unconfigured” vocal tract, how can vowel-like qualities approximating those of /u/ be achieved?

Here, we investigate whether vowel-like acoustic properties comparable to those of the human close back rounded vowel /u/ can be achieved via acoustic tube models designed to emulate chimpanzee vocal tracts. The logic assumed here is that vocal tract resonances (hereafter formants) of actual* hoo*’s can be reconstructed by recreating airflow through a series of narrow tubes roughly equal in length to the vocal tract length (VTL) of the original vocalizer. Where tube sequences result in comparable resonance values, we assume that the sequence of tubes roughly recreates (one possible alternative of) the shape of the vocal tract of the vocalizer in terms of the *F*_1_–*F*_2_ dispersion. The work described here is mainly acoustic in nature; we do not claim that the proportions used are realistic with respect to great ape vocal tracts.

## Methods

### Acoustic properties of chimpanzee* hoo*’s

**Fig. 2:**
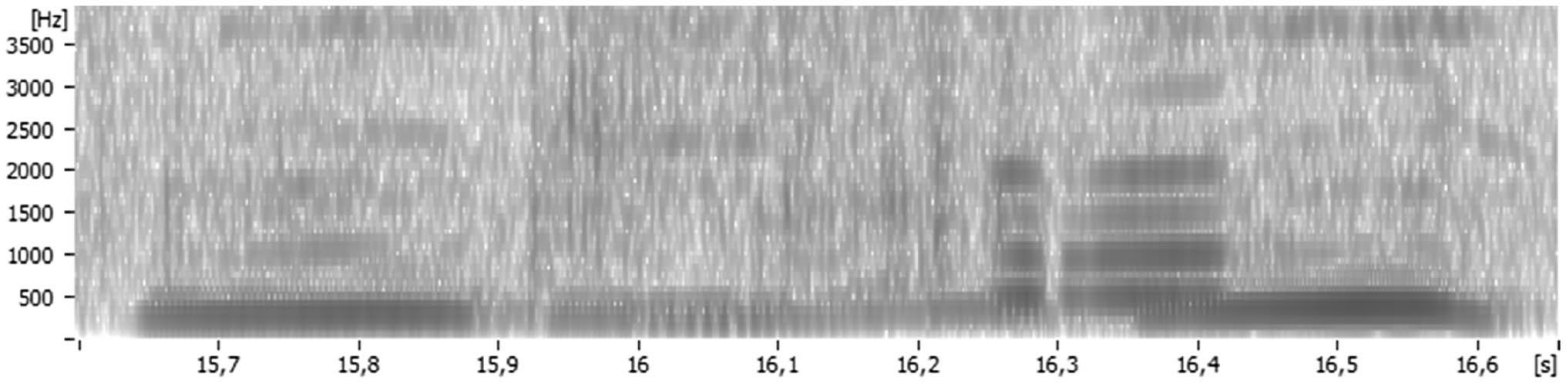
Spectrogram (300 Hz) of a chimpanzee* hoo*. The phonetic properties of the calls consistently showed two prominent spectral peaks corresponding to /u/-like resonance dispersions, with the first one at around ~ 300 Hz and the second one at around ~ 1 kHz. A clear example of this is visible at 16.3 s. Spectrogram rendered in Sopran. Note that in chimpanzee hoo's, the fundamental frequency often overlaps with *F*_1_. Our estimates are consistent with those reported elsewhere (Grawunder et al., 2022)

To determine whether the acoustic and perceptual properties of chimpanzee* hoo*’s indeed overlap with those of human back vowels, we sampled, segmented, and analyzed a small selection of chimpanzee* hoo*’s (*n* = 8; three individuals). The recordings (ML163620, ML163621, ML163626; Macaulay Library, Cornell Lab of Ornithology, Ithaca, NY) were made by van Plooij et al. (2015). The dataset metadata include quality ratings, and only recordings of the highest quality were curated for the study. Furthermore, although bouts of pant–hooting are performed with both ingressive and egressive phonation, to determine acoustic similarity to human vowels, all sampled hoot segments were egressive (i.e., the airflow was expiratory). Targeted calls were sampled from the “introduction” and “build up” phases of pant–hooting (hereafter *hoo*’s) (Fig. 2), as at later phases the calls transitioned into high-frequency screams. All of the sampled calls were produced by males. The average length of a segment is 0.61 s (SD = 0.31).

Analyses of recordings were conducted according to the primate quasi vowel (PREQUEL) protocol (Ekström et al. 2023). Formants were estimated via visual inspection as unsupervised methods such as linear predictive coding may skew formant estimation when applied to chimpanzee vocalization data (Ekström 2023d). Estimates were corroborated with output from the Madde additive vowel synthesizer (Tolvan.com) matched for fundamental frequency (*f*_0_) (perceived as pitch). The average observed formants (*F*) were *F*_1_ = 358.75 (SD = 56.93) and *F*_2_ = 896.25 (SD = 133.04), which indeed overlap with those of human back vowels (Table 1).

**Table 1:** Formant (*F*) data for human /u/ (Peterson and Barney 1952) and chimpanzee* hoo*

	Human /u/			Chimpanzee *hoo*
	Male	Female	Child	
*F*_1_				
Mean	307.36	377.86	432.37	374.44
SD	50.01	46.76	87.48	67.02
*F*_2_				
Mean	875.97	960.57	1193.33	896.25
SD	155.46	171.46	274.61	133.04

### Computational approach

We used the Tuben python package (Ekström and Beskow 2023) adapted using the modeling approach developed by Liljencrants and Fant (1975) to simulate vocal tracts. The method is based on the circuit theory established by Fant (1960) and derives vocal tract resonance frequencies from area functions of tube representations of vocal tracts based on volume–velocity glottal lip transfer. The assumption at the heart of this method is that the voice “source” from the larynx will be reliably “filtered” (Fant 1960) given the dimensions of the tract. The physiological basis of the source, or phonation, is well preserved across primates (Negus 1949). Changing the vocal tract configuration by moving one or more of the articulators (tongue, velum, etc.) affects the resulting vowel quality. Broadly, /i/ (the vowel in “see”), for example, can be modeled as a relatively open tract (corresponding to an open pharynx) but with constriction close to the end of the tube (corresponding to a tongue tip or blade close to the anterior hard palate). The code supplied in the original publication has been converted into Python and is publicly available (https://github.com/jbeskow/tuben). The code was adapted to systematically generate sequences of tubes based on variations of five parameters (Table 2). Vocal tract tube models in which rigid rounded structures are assumed cannot realistically capture the intricate acoustic significance of the properties of flesh, cartilage, bone, and viscosity that make up actual vocal tracts. However, when properly implemented, they allow for the variation of parameters that are likely to affect the properties of filtered voice signals. The speed of sound was set to 35 m/s (for a room temperature of ~ 20 °C). To avoid any fine-tuning, and to preserve the integrity of the experiments, no further changes were made to the models. For the mathematical bases of the program, including properties of the walls and transfer functions, see Liljencrants and Fant (1975) and the publicly available code.

**Table 2:** Parameters employed in the simulations

Parameter	Range	Increment
Length of lip protrusion (cm)	0.2–3.8	0.2
Length of air sac (cm)	1–2	0.5
Area of air sac (cm^2^)	1–30	1
Area of lip passage (cm^2^)	0.2–1	0.2

### Model parameters

#### Vocal tract length

To our knowledge, the only reported VTLs for adult chimpanzees are those of Nishimura (2005), who estimated a VTL of 18.12 cm for one adult male (the values were computed by adding the lengths of horizontal and vertical vocal tract sections). Accordingly, we set the VTL parameter in our computational models to 18 cm. The area of the VTL was held constant at 1 cm.

#### Lips

The length of a great ape’s vocal tract can be apparently extended, and its opening narrowed, by movement of the lips (Lieberman 1968; Grawunder et al. 2022). Nonhuman great apes possess larger, fleshier lips than humans, which can even be used for object manipulation (Rogers et al. 2009; Iwasaki et al. 2019). Lip protrusion is evidently employed by chimpanzees in the production of a *hoo* (Parr et al. 2005; Grawunder et al. 2022). Extension via lip protrusion of up 3.8 cm (in increments of 0.1 cm) was assumed for the computational models. (N.B. As the total extendable length of a chimpanzee lips is, to the best of our knowledge, unknown, as is lip length in the production of a hoot, the parameter values used here are provisional and simplistic. However, if these data do become available, they could easily be incorporated into iterations of the described models, which would improve their goodness of fit.) Lip rounding, which is employed in articulation by human speakers in vowel production, and by chimpanzees to produce* hoo*’s (Grawunder et al. 2022), was varied between 0.2 cm^2^ and 1 cm^2^ (in increments of 0.2 cm^2^) in our models. Length was kept constant at 0.2 cm, for a total elongation of the VTL of 4 cm.

#### Laryngeal air sacs

The acoustic and functional properties of laryngeal air sacs, which are found in most nonhuman primates (Negus 1949; Hewitt et al. 2002), are not well understood. Increased knowledge about these structures and why they have been selected against in human evolution could provide valuable insight into the evolution of human speech (De Boer 2012). Chimpanzee air sacs are of the lateral ventricular type, extending from laryngeal ventricles above the vocal folds before fusing in the ventral neck region and then expanding caudally and/or cranially (Hewitt et al. 2002; Hayama 1970). Acoustic modeling of air sacs was performed by De Boer (2009, 2012; see also Gautier 1971), and the possible function of these structures was discussed by Lieberman 2006). de Boer (2009, p. 297) stated that “… [an air sac] shifts up the oral tract’s resonances below approximately 2000 Hz, and shifts them closer together.” However, to our knowledge, the acoustic effects of air sacs have only been explored for nonhominid primates such as howler monkeys and gibbons (De Boer 2012), not for great apes (though both gibbon and chimpanzee air sacs are of the laryngeal ventricular type), or rhesus macaques (Hilloowala and Lass 1978), nor have they been explored using tube vocal tract models. Chimpanzees, however, unlike these species, may be capable of significantly extending their oral tract by protruding their lips (Grawunder et al. 2022), which may have an effect on formants.

**Fig. 3a–e:**
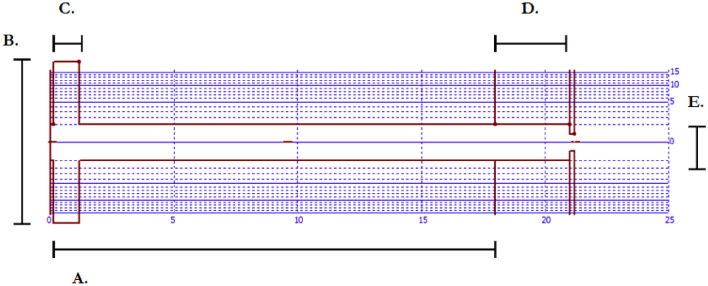
An example vocal tract configuration, with total length shown by the *x*-axis and area (logarithmic) by the *y*-axis. **a** Vocal tract length, **b** area of air sac simulacrum, **c** length of air sac simulacrum, **d** length of lip protrusion, and **e** area of lip opening. Cross-sectional area of each segment is denoted by its length and area. [Image rendered using Wormflek software (Johan Liljencrants, KTH Royal Institute of Technology)]

In our models, an attempt was made to examine something of the influence of the air sacs by assuming a narrow constriction of 0.125 cm before the large open cavity (air sac) (Fig. 3). The “length” of the air sac was varied between 1 and 2 cm, at increments of 0.5 cm. The area of the sac was systematically varied between 1 cm^2^ (i.e., the absence of an air sac; the air sac was considered to be a uniform tube, except when lip protrusion was varied) and 30 cm^2^, in increments of 1 cm (Table 2). These values are simplistic and tentative; the purpose of the exercise was to give an indication of the influence of the air sac simulacrum on call acoustics—we do not claim that the dimensions used here are realistic. Future studies could use the methods presented here to investigate this relationship more thoroughly, for example, by narrowing and widening the tube, or by decomposing it into multiple sections of variable length and area.

### Synthesis and perception

To assess the validity of the predicted formants, the *F*_1_–*F*_2_ dispersion was synthesized for the closest fit for observed chimpanzee* hoo*’s. The syntheses were computed using the phonTools package for R (Barreda 2015) with the vowelsynth function, based on Klatt (1980). The length of the synthesized sound was held constant at 2 s, and *f*_0_ was held constant at 100 Hz to preserve vowel quality, which degrades at higher *f*_0_ (e.g., Ryalls and Lieberman 1982).

## Results

### Mapping predicted formants

**Fig. 4:**
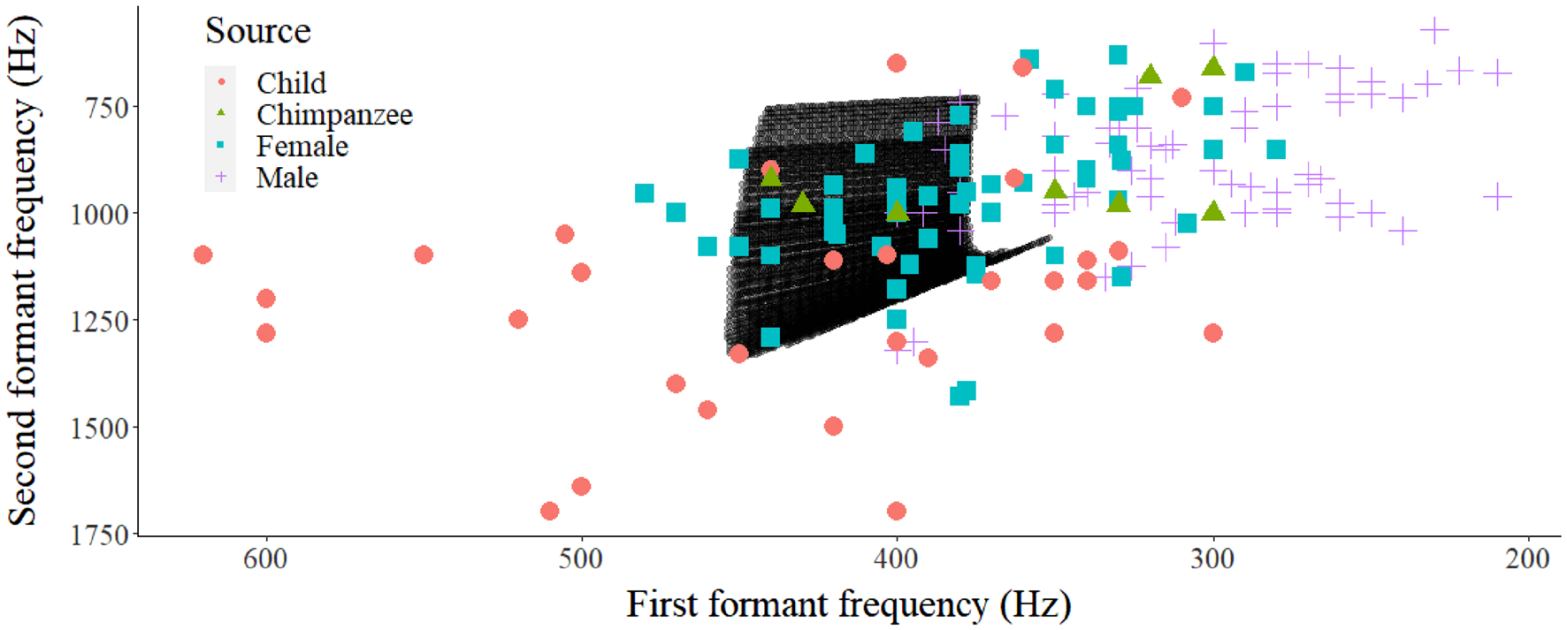
Results of simulations. By applying the assumptions outlined in the text, the fit for characteristics of the human back rounded vowel /u/ as spoken by male and female adults and children increased (Peterson and Barney 1952)

**Fig. 5:**
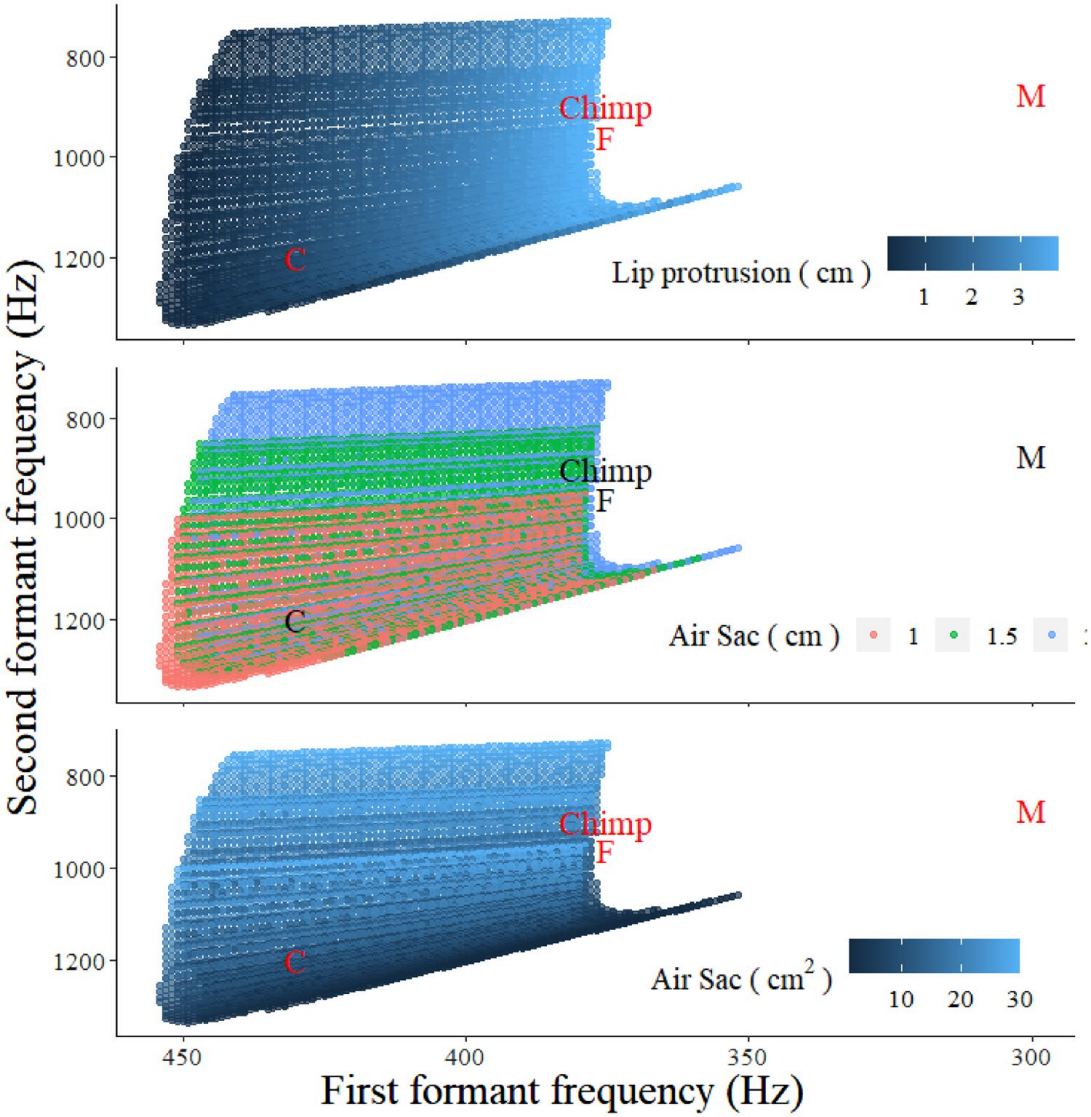
In our simulation, values of *F*_1_ are highly contingent on the length of the lip protrusion segment, while *F*_2_ shifts downwards with larger, more voluminous air sac simulacra. Plotted simulation data are identical for Figs. 4 and 5. The graph has been re-scaled to illustrate differences between categories of data. Mean *F*_1_–*F*_2_ coordinates for /u/ spoken by adult males (*M*), females (*F*) and children (*C*) (from Peterson and Barney 1952) and chimpanzee* hoo*’s (*Chimp*) are superimposed. Illustrations created with ggplot2 (Wickham, 2011)

Vowel formant data for /u/ from children and adult male and female speakers were obtained from Peterson and Barney (1952) for comparison (Table 1). *F*_1_–*F*_2_ dispersions overlapped with those of /u/ for multiple models (Figs. 4, 5). There was a general trend in the simulation data which illustrated that combinations of longer vocal tract, larger air sacs, and greater lip protrusion shifted the predicted formants such that the vowels assumed qualities that were more indicative of back vowels. All simulation data are publicly available from GitHub.

### Perception

We perceived the quality of the resultant vowel as that of the close back rounded vowel /u/, which is consistent with previous reports (e.g., Peterson and Barney 1952; Fant et al. 1969; Catford 1988). The quality of the synthesized vowel is publicly available at https://github.com/evofant/chimpanzee_u.

## Discussion

Chimpanzees are probably unable to articulate the human-like close back rounded vowel /u/ because of limitations to their articulatory morphology (Negus 1949; Lieberman 1984, 2012; Takemoto 2008; De Boer and Fitch 2010). Here, we computed vocal tract area transfer functions for a series of tubes with the intention of roughly simulating a chimpanzee’s vocal tract via an unconfigured vocal tract. Formant frequencies approximating those of back vowels were achievable by assuming the presence of an open cavity immediately after the source of the voice (an intentionally simplistic simulacra for a laryngeal air sac), followed by a uniform tube, with protruding “lips.” While being acoustically and perceptually comparable to human back vowels, the results of the present study indicate that chimpanzee* hoo*’s may reflect distinct vocal tract shapes that are not readily employed by human speakers. The apparent /u/-like quality of chimpanzee* hoo*’s to a human listener, thus, may result from an acoustically fortuitous phenomenon, while also reflecting disparate articulatory states between species.

### Limitations

It is important to note that this work is principally acoustic, and that the procedure employed is based on various assumptions regarding vocal tract shapes and transfer functions that do not necessarily reflect those of an actual chimpanzee vocal tract. Validation of our findings is contingent upon obtaining reliable estimates of these properties. These measurements would also allow for fine-tuning of the modeling approach described here. More importantly, however, our models, being composed of only five tubes characterized by two parameters—length and an area transfer function—likely do not capture the inherent acoustic relationships of an actual chimpanzee’s vocal tract. For example, while we assumed a VTL of 18 cm, circumstantial evidence suggests that the vocal tract may be actively elongated while a chimpanzee vocalizes. First, the vocal tract may be elongated via select articulatory gestures, including lowering of the larynx and lip protrusion. Note, however, that while larynx lowering per definition cannot increase the phonetic range of the animal (see e.g., De Boer and Fitch 2010; Lieberman 2012), the resulting elongation of the vocal tract shifts down formants, possibly facilitating properties comparable to those of back vowels (which are characterized by low *F*_1_ and *F*_2_). Furthermore, earlier modeling work suggests that air sac volume, neck dimensions, and mass of the walls are the most significant factors affecting resonance (De Boer 2012). It is important to note that the approach presented here only allows for changes in the first. Most air sacs have soft walls, which allows them to readily change shape and thus volume (Hewitt et al. 2002). As chimpanzee lips are large and fleshy, we cannot rule out the possibility that there are an impediment to vocalization, the effects of which would not be captured by our method. Thus, the results presented here should be interpreted with caution. The comparison of audio recordings and VTL estimates for the same animal would substantially narrow down to what degree the assumptions made here are appropriate. The primary goal of this exercise was to stimulate further research on great ape articulation—the least understood aspect of great ape vocalization and behavior.

### Future directions

The limited availability and quality of relevant physiological data have long been constraints to investigations in phonetic sciences. The magnitude of this problem is even greater when the vocalizer under investigation is a nonhuman animal that cannot follow instructions or agree to invasive procedures. To date, little research has been conducted on the vocal tract dynamics of actual chimpanzees (but see Grawunder et al. 2022). The results of the present study may be useful for the prediction and validation of these types of data, and could also serve to improve the quality and precision of simulations. Importantly, our results suggest that vowel-like sounds similar to human vowels may be achievable in chimpanzees, and that they may not, given the disparate vocal tract configurations of the two, indicate evolutionary continuity per se.

Our results indicate that unconfigured vocal tracts—which are characterized by a narrow pharyngeal cavity and flat tongue, such as those found in extant nonhuman great apes, including chimpanzees—may, given sufficient lip extension and rounding, achieve vowel qualities comparable to those of human back vowels, without comparable articulation needed to produce them. However, to more realistically model chimpanzee articulation, we would specifically like to be able to compare outcomes against great ape hoot calls. In the present study we were limited to using a small selection of calls, but ultimately, we would like to try to reverse-engineer various aspects of great ape call repertoires. Our approach may also enable further investigation of a variety of related phenomena inherent to great ape vocalization. For example, by allowing the algorithm to be fine-tuned, our computational approach may also enable researchers to test hypotheses on the functioning of air sacs (Negus 1949; De Boer 2009; Lieberman 2006). Our air sac simulacra are highly simplified, and future efforts that employ the method used here may—based on the same computational principles—be able to derive an optimal simulacrum that mimics the effects on filtered signals reported by De Boer (2012). Finally, in the present study, we evaluated the vowel quality of synthesized vowels aurally, and have made the relevant files publicly available. To our knowledge, however, ape vowel-like calls only rarely been presented to human listeners as part of a perception experiment (Ekström et al., 2023). This is yet another potential avenue of research. If chimpanzee vowel-like qualities were shown to be more inconsistently perceived as such, compared with human vowels, this may provide additional clues as to the evolution of phonetic capabilities in ancestral hominids. Finally, increasing our understanding of great ape airway and vocal tract dynamics by using the methods presented here may also be of benefit to animal welfare projects.

### Conclusion

It is unlikely that chimpanzees can achieve human-like articulation of close back rounded vowel /u/. The preliminary data presented here indicate that vowel qualities similar to those of human back vowels are achievable with a vocal tract consisting of a largely uniform tube, given sufficient lip extension. Thus, while acoustically and perceptually comparable to human back vowels, chimpanzee* hoo*’s may reflect vocal capabilities that are distinct from those of modern humans. Comparative work is needed to test and verify this by collecting data on a wider selection of in vivo vocalizations. The results of our simulations are a tentative indication that nonhuman great apes, while limited with regard to producing human-like speech sounds, may nonetheless possess a flexible articulatory apparatus that enables movements of the lips that cannot be achieved by humans. Our results illustrate that the apparent similarity of great ape calls to human vowels need not reflect evolutionary relationships per se.

## Data Availability

Not applicable.
